# Psychosocial rehabilitation of individuals with schizophrenia: a scoping review protocol

**DOI:** 10.1186/s13643-022-01901-y

**Published:** 2022-02-19

**Authors:** Oyeyemi Olajumoke Oyelade, Nokuthula Gloria Nkosi-Mafutha

**Affiliations:** 1 Department of Nursing Education, School of Therapeutic Sciences, University of the Witwatersrand, Park Town, Johannesburg, South Africa; 2 Department of Nursing Science, Faculty of Basic Medical Sciences, Obafemi Awolowo University, Ile-Ife, Osun-State Nigeria

**Keywords:** Chronic mental illness, Schizophrenia, Rehabilitation, Global and African health

## Abstract

**Background:**

The psychosocial rehabilitation of an individual with mental illness is an evidence-based approach to reducing the burden of the illness and the associated stigma globally. Specifically, in Africa, it has promising scope for African life and the African economy. Psychosocial rehabilitation is described as a set of approaches that aim to assist an individual in achieving restoration from a state of dependency caused by schizophrenia to a state of being an independent decision-maker. However, there seems to be a dearth of literature and implementation of psychosocial rehabilitation in Africa. Therefore, it is necessary to map studies on how psychosocial rehabilitation is conducted for people living in Africa with the most chronic form of mental illness, schizophrenia.

**Methods:**

This study will adopt the Arksey and O’Malley scoping review framework to search and compile relevant studies. This process will involve three steps: title screening, to be performed solely by the principal investigator, followed by abstract and full-text screening, to be performed independently by two reviewers (the principal investigator and co-investigator). Rayyan QCRI, a systematic reviews web app, will be used for tracking the screening records, and data charting form will be used to extract basic data of included studies. The risk of bias in the articles identified for screening will be assessed by the Mixed Method Appraisal Tool (MMAT). Finally, the content analysis of the screened studies will be performed with NVivo.

**Expected outcome:**

This study has the likelihood of revealing a research gap in psychosocial rehabilitation approaches and methods. The review results will constitute part of the available evidence that the researchers aim to adopt in the broader part of the project, which aims to develop implementation strategies for the psychosocial rehabilitation of chronic mental illnesses, specifically schizophrenia, in Sub-Sahara Africa. The implementation process also encompasses disseminating the findings of this review to stakeholders, which will enhance their knowledge of the current state of Sub-Saharan Africa and may stimulate support for the implementation of rehabilitation strategies.

## Background

Schizophrenia has been declared the most chronic mental illness and the mental illness that attracts the most self-blame in Sub-Saharan Africa, as opposed to alcoholism and drug addiction, which attract the most self-blame in Western countries [1]. The belief that schizophrenia is self-inflicted affects the care-seeking pattern for those affected in Sub-Saharan Africa. Most individuals are taken to traditional healers or left to suffer due to the belief that the wrath of the gods victimizing the individual will stop once the gods are satisfied [2]. Traditional healers in Sub-Saharan Africa declare that mental illness is treated through concussion, divination, and rituals to appease the gods [3]. This suggests that individuals with mental illness are liable to chronicity and have an increased burden from delayed treatment in Sub-Saharan Africa. Mental illness constitutes 14% of the global burden of disease and 30% of the burden of non-communicable diseases [4, 5], with schizophrenia being the most chronic form of the illness, contributing to approximately 7% of the global burden [6]. However, there is a dearth of resources for estimating the burden of schizophrenia in Sub-Saharan Africa. The global burden of mental illness is considered significant since it has the highest rate of disabling effect (regarding low or no productivity) on individuals globally [7, 8]. Specifically, in Africa, mental illness accounts for 18% of a life lived with disability [9]. If an individual has a mental illness for 10 years, the person will be utterly dependent for 730 days (2 years) of his/her life. Moreover, there is a 40–60% chance of premature death in people with schizophrenia [10].

The disabling effect of schizophrenia not only affects individuals but also affects their families and health practitioners. The burden on families stems from monitoring the patient’s hygiene, the use of medication, comportment at social gatherings, follow-up with health care providers, and the need to deal with mood, cognitive and behavioural changes [11–13]. The burden of care on families with schizophrenia causes emotional distress in family members and increases their chances of also developing schizophrenia or other forms of mental illness [14]. Specifically, in Africa, the family burden is compounded by family labelling, besides the delayed treatment reducing the speed of recovery. In addition, members of the community segregate families of individuals with schizophrenia by refusing to allow them to live within their neighbourhood [15].

Additionally, community members refuse to enter into marital relationships with the siblings of individuals with schizophrenia even when those siblings do not have the ailment [16]. This implies that individuals with schizophrenia and their families have little or no chance of living within the community or getting married and procreating in Sub-Saharan Africa. This might also be responsible for locking them indoors and being secretive about having a relative with schizophrenia [17]. According to Orjiakor and colleagues, societal behaviour towards individuals with schizophrenia causes challenges with social interpretation and violence in Sub-Saharan Africa, which constitutes another burden [18].

The burden of schizophrenia on health practitioners is related to the scarcity of human resources, which the World Health Organization [19] has declared as an important requirement for quality care in mental health. Health practitioners engage in diverse methods to achieve recovery. These methods involve pharmacological (antipsychotics) and non-pharmacological (family therapy, individual therapy, group therapy, physical therapy, and occupational therapy) methods, but these methods alone do not reduce the burden [20–23]. The World Health Organization recommends psychosocial rehabilitation [24], for reducing the burden of mental illness. Psychosocial rehabilitation (PSR) is described as a set of approaches that aim to assist an individual in achieving restoration from a state of dependency caused by schizophrenia to a state of being an independent decision-maker [25, 26]. It encompasses promoting quality of life. It is considered the most significant health care action for facilitating and sustaining the recovery of persons with mental illness, including schizophrenia [27, 28]. PSR promotes the holistic view of the person by providing vocational, educational, residential, social/recreational and personal adjustment services [29, 30].

Evidence from global research shows that over 200 (251–261) mental illness disability-adjusted life years (DALYs p.a./1 million pop.) may be averted through psychosocial rehabilitation interventions compared with 149–160 DALYs averted through medication alone [7, 9, 19, 31]. Specifically, the World Health Organization emphasizes that PSR should be contextually relevant for practice in each country [24]. Despite the available evidence about the effectiveness of PSR, there seems to be a dearth of PSR implementation in Sub-Saharan Africa [26, 28, 32]. Global studies reveal that only two African countries (Botswana and South Africa) have the standard psychosocial rehabilitation practice guide [19, 33]. Additionally, through the Mental Health Action Plan 2013–2020, the World Health Organization has declared a need for increased service coverage through integrated and responsive rehabilitation [19]. Over the decades, high-income countries have managed to implement context-specific psychosocial rehabilitation. However, Sub-Saharan African countries still struggle with the implementation of rehabilitation for individuals with schizophrenia. The struggle is defined as a lack of literature, scientific know-how and implementation of psychosocial rehabilitation in Africa [26, 28, 32]. However, some studies reveal that some countries in Sub-Saharan Africa engage in rehabilitation despite the identified limitation [26, 28, 32].

PSR implementation in high-income countries has led to status disclosure, reduced institutionalization, self-awareness, increased productivity and reduced stigma. This result was contrary to experiences of Sub-Saharan African countries of labelling and intense stigmatization [34, 35]. This study intends to scope and review the available evidence of rehabilitation in Sub-Saharan Africa as a first step to assessing practice strategies geared towards instituting scientific implementation strategies at a broader scope of this study. Though there are systematic reviews on rehabilitation in Sub-Saharan Africa, their focus was on policy implementation and economic burden warranting rehabilitation and not how it is done, which is what this study purposed to do [22, 36–38]. Also, the available reviews from western countries were not solely focused on Sub-Saharan Africa, and their focus was on access and availability of rehabilitation strategies and not how it is done [23, 39–41].

### Review objectives

The objectives of the scoping review are as follows:

- To explore the existing evidence on psychosocial rehabilitation of schizophrenia and its approaches in Sub-Saharan Africa
- To examine the opportunities available to individuals with schizophrenia and the outcomes of studies or exposure to the available opportunities in Sub-Saharan Africa
- To refine future PSR research questions, to fill gaps in evidence on rehabilitation research in Sub-Saharan Africa

## Methods

### Design

The protocol is for a scoping review of studies on the PSR of individuals with schizophrenia in Sub-Saharan Africa. The review will be guided by the Arksey and O’Malley framework [42]. This framework involves the following sequence:

1. Setting the research question
2. Identifying the relevant studies
3. Selection of studies
4. Data charting
5. Collation, summary generation and report writing

### Setting the review question

The review is underpinned by the following question: what work has been done in the psychosocial rehabilitation of people living with schizophrenia in Sub-Saharan Africa?

The sub-questions are as follows:

- What is the existing evidence on the psychosocial rehabilitation of schizophrenia and its approaches in Sub-Saharan Africa?
- What was the expressed goal of the individuals with schizophrenia in Sub-Saharan Africa, and what difference did participating in rehabilitation make in their life generally and specifically in the achievement of their life goal?
- What are the existing gaps in psychosocial rehabilitation research in South Sahara, Africa?

### Identification of the relevant studies

#### Search for the relevant studies

Primary data (from published and unpublished studies) that address the research question will be identified for possible review. The searches for published literature will be performed using the LibGuides online bibliography and ten databases (the listed databases (PubMed, Scopus and Sabinet) and selected databases under the listed platforms, ProQuest (PsycINFO (Psychological Information), Embase, Medline) and EbscoHost (Academic Search Complete, ERIC, Health Source, Psychology and Behavioral Sciences Collection) will be consulted. The following sites will also be consulted for unpublished literature: Google, Google Scholar, African Journals Online, and HINARI. In addition to the electronic search of databases, the search approach will include checking the references of included studies and related reviews and contacting experts in searching grey literature sources such as conference proceedings. The search terms will include psychosocial rehabilitation AND rehabilitation AND schizophrenia AND Sub-Saharan Africa. Three library experts from three different countries were consulted. One was a medical school librarian, and two were librarians from schools of public health to guide search terms for “schizophrenia”, “rehabilitation” and “Sub-Saharan Africa” (the full list of variations on the search strategy is available in the search sample below). According to reports of library experts, schizophrenia can also be conceptualized as a chronic mental illness, while rehabilitation is conceptualized as productivity. In alternate words, Sub-Saharan Africa is also regarded as Black Africa. The variant terms will also be considered in the study. Duplicates will be removed, and the eligibility criteria will be applied to select studies that will be reviewed in the study.

#### Relevant studies search guide (population concept and context (PCC))

The 2015 study population concept and context (PCC) scoping review framework by Arksey and O’Malley, shown in Table 1, will be used to determine the potentially relevant studies for inclusion in the review.

**Table 1:** PCC framework

Criteria	Determinants		
	Main concept	Alternate keywords	Subject headings
**P**opulation	Schizophrenia (individuals with schizophrenic disorders)	Chronic mental illness OR chronic psychiatric illness OR chronic insanity OR chronic mental disorder OR dementia praecox OR Schizophrenic psychosis OR schizophrenic disorder OR schizophreniform disorder OR schizoaffective disorder	Individuals with schizophrenia disorders
**C**oncept	Rehabilitation	Productivity OR re-integration OR reformation OR rehabilitation OR recovery functioning OR halfway housing OR re-establishment OR occupational skills OR vocational skills OR independent living skills OR reclamation OR improvement	Psychosocial rehabilitation
**C**ontext	Sub-Saharan Africa	Sahara Africa OR Black Africa OR southern Africa OR Africa OR south of the Sahara OR Africa OR developing countries OR low economies countries OR third world countries OR low income countries	Africa
	Sub-Saharan African countries	Angola, Benin, Botswana, Burkina Faso, Burundi, Cabo Verde, Cameroon, Central African Republic, Chad, Comoros, Congo, Dem. Rep., Congo, Rep., Cote d’Ivoire, Equatorial Guinea, Eritrea, Eswatini, Ethiopia, Gabon, Gambia, Ghana, Guinea, Guinea-Bissau, Kenya, Lesotho, Liberia, Madagascar, Malawi, Mali, Mauritania, Mauritius, Mozambique, Namibia, Niger, Nigeria, Rwanda, Sao Tome and Principe, Senegal, Seychelles, Sierra Leone, Somalia, South Africa, South Sudan, Sudan, Tanzania, Togo, Uganda, Zambia, Zimbabwe	Sub-Saharan Africa

### Selection of studies

#### Eligibility criteria (inclusion and exclusion)

A specific duration will guide the search strategy for this review, and studies before 2000 will not be considered for review because the global advocacy of the mental health burden and rehabilitation started in 2000 [24]. Specifically, studies from 2000–2021 will be included. Studies that are not available through the South African inter-university library system will be searched with the assistance of the librarians of the Consortium for Advanced Research Training in Africa (CARTA) and African Population and Health Research Center (APHRC). In addition, the abstract and other available information on studies not available within all these possible facilities will be considered. The context of the studies will also be considered, and studies generated outside Sub-Saharan Africa will be excluded. There will be no language barrier in study selection as both studies are written in English, and other languages will be included. However, there may be a tendency to misinterpret studies written in other languages, but direct and reverse translation will be arranged from institutionally recognized language expect to avoid misinterpretation. Also, the journals will be consulted to inquire if translated copies are available.

#### Searching and screening

This stage comprises three phases. The first phase is the search stage which consists of 1) developing and refining a search in one database, 2) translating the search to all the other databases and 3) importing/gathering the results in software (Endnote) to remove duplicates. The second and the third phases are the screening phases which involve a title, abstract and full article screening using Rayyan QCRI, a systematic reviews web app, for screening and tracking the screening records.

Phase 1: This phase is operationalized in this protocol as pilot testing. Existing reviews on schizophrenia and psychosocial rehabilitation were consulted to elaborate the searches for each concept, and the findings are presented in Table 2. In addition, the search terms were piloted as indicated in Table 2 to ascertain the appropriateness of the search terms. The PubMed search will be translated to all databases in the full review, and Rayyan QCRI will be used to keep a record of studies retrieved.

**Table 2:** Piloted database search results

Date of search	Keywords	Database	Publication retrieved	Link	Search terms
03 November 2021	Schizophrenia AND Rehabilitation AND Sub-Sahara	PubMed	618	https://pubmed.ncbi.nlm.nih.gov/?term=longquery54edd454fcf4884e7dcc&filter=dates.2000-2021	(schizophrenia OR “chronic mental illness” OR “chronic psychiatric illness” OR “chronic insanity” OR “chronic mental disorder” OR “Schizophrenic disorders” OR “dementia praecox” OR “Schizophrenic psychosis” OR “schizophreniform disorder” OR “ schizoaffective disorder”) AND (rehabilitation OR productivity OR re-integration OR reformation OR rehabilitation OR “recovery functioning” OR “halfway housing” OR re-establishment OR “occupational skills” OR “vocational skills” OR “independent living skills” OR reclamation OR improvement) AND (“Sub-Sahara Africa” OR “Black Africa” OR “southern Africa” OR “Sahara Africa” OR Africa OR “south of the Sahara” OR “developing countries” OR “low economies countries” OR “third world countries” OR “low income countries” OR Angola* OR Benin* OR Botswana* OR “Burkina Faso*” OR Burundi* OR “Cabo Verde*” OR Cameroon* OR “Central African Republic*” OR Chad* OR Comoros* OR Congo * OR “Democratic Republic of Congo*” OR “Republic of Cote d’Ivoire*” OR “Equatorial Guinea*” OR Eritrea* OR Swaziland* OR Ethiopia * OR Gabon * OR Gambia * OR Ghana* OR Guinea* OR “Guinea-Bissau *” OR Kenya * OR Lesotho* OR Liberia* OR Madagascar* OR Malawi * OR Mali* OR Mauritania* OR Mauritius* OR Mozambique* OR Namibia * OR Niger* OR Nigeria* OR Rwanda* OR “Sao Tome and Principe*” OR Senegal*OR Seychelles* OR “Sierra Leone*” OR Somalia* OR “South Africa*” OR “South Sudan*” OR Sudan* OR Tanzania* OR Togo* OR Uganda* OR Zambia* OR Zimbabwe*)

The table above shows the summary of studies generated by a database search. Limit setting: year (2000–2021)

Phase 2: This involves the title screening of the studies from each database. The keywords with the variant search terms as displayed in Table 2 will be input into each database without modification for consistency of the search terms. This stage will only exclude studies that do not include the schizophrenia population or rehabilitation concept. The titles of the resultant pool of studies will be assessed by one of the reviewers (the principal investigator). Titles with the population and concept of the study will be selected. It is important to assess the population because the researcher has a population of interest: individuals with schizophrenia. Therefore, the researcher also has a concept of interest, that is, rehabilitation. Assessing the titles one by one for these two indices, schizophrenia and rehabilitation is necessary to streamline the selected titles to titles of interest. The alternate word for such a concept will also be considered in the title to avoid excluding potentially relevant articles that do not contain schizophrenia and rehabilitation.

The eligible titles will be exported into a storage file (Rayyan QCRI, a systematic reviews web app). The records of the titles screened will be documented as shown in Table 3.

**Table 3:** Electronic records of the titles screened

**Keyword**	**Database used**	**Number of studies retrieved**

The records of the titles screened will be kept in three columns. The first column consists of the keywords inserted into the search space. The second column will record each database where the search was conducted. The last column consists of the number of studies retrieved from each database.

Phase 3: The abstract and full-text screening will be performed by two screeners (the principal investigator and co-investigator) using Rayyan QCRI software. Tables 4 and 5 represent the format of tabulation of both screenings. The two reviewers will conduct an independent screening of the abstracts and full texts and compare the results. Rayyan QCRI, a systematic reviews web app generated by the principal investigator during title screening, will be shared with the co-investigator for co-screening. There are discrepancies between the two screeners; they will be resolved by critically looking at the areas of differences together for resolution and agreement. A third screener will be incorporated in situations where agreement is not reached, and the three reviewers will screen independently. Of the three results, two similar results will be considered. The flow of studies found by searches screened and included in the final review will be reported in a PRISMA flow chart and reported according to the PRISMA 2018 guidelines for scoping review [43].

**Table 4:** Abstract screening table

S/N	Author and date	Is the study from Sub-Sahara Africa?	Does the study include individuals with schizophrenia?	Does the study include psychosocial rehabilitation?	Initial of the screener	Report of screener A	Report of screener B
		Yes or no	Yes or no	Yes or no			

The eligibility for inclusion of studies with screened abstract will be to have at least two “yes”(es) in any of the three sections that bear “yes” or “no” responses under abstract screening as shown in Table 4*.*

**Table 5:** Full article screening table

S/N	Author and date	Study title	Are there formal or informal personnel involved in the process of rehabilitation?	What is/are the study setting(s)?	What is/are the pattern(s) of communication in the rehabilitation setting?	Does the study include feedback on the effectiveness of the rehabilitation?	Initials of the screener
			Yes or no to informal personnel			Yes or no	

The eligibility of an article for inclusion will be to have a “yes” in any of the two columns that require “yes” or “no” responses, as shown in Table 5.

### Charting of data

The data charting form indicated in Table 6 will be used to assess the basic information in each of the studies. This will be done by two independent reviewers using NVivo. The variables in the charting form are suitable for answering the research questions and will be modified as deemed fit during the charting process.

**Table 6:** Data charting form

Author and date of publication	
Title of the study	
The aim of the study	
Country of the study/location	
Method/methodology	
Population/participants	
❖ Formal and informal health care providers	
❖ Individuals with schizophrenia	
Research question/objective	
Study setting/context	
❖ Clinical	
❖ Community	
Study design	
Data collection methods	
Study limitations/challenges	
Data analysis	
Study outcomes/results	
Significant findings	
Conclusions/recommendation	

### Collation, summary generation and report writing

The studies screened for inclusion in this review will be collated in NVivo. The content analysis approach will be used to analyze each of the studies in NVivo. Themes will be derived from the categories of meaning units of each of the studies. The theme derivation will evolve from the meaning units in each category. The meaning units are information derived from the study based on the review objectives, and it will form the basis of the report on rehabilitation. The report will be written based on the themes generated, which will inform the conclusion and recommendations of the review.

#### Risk of bias

The risk of bias in each of the identified articles will be assessed using the Mixed Method Appraisal Tool (MMAT) version 2011. The tool will serve as a scientific assessment instrument for checking the rigour of qualitative methodologies, the validity of quantitative instruments, the suitability of methodologies and the appropriateness of mixed-method approaches [44]. The principal investigator will use the tool to assess the designs, data collection methods, analytical approaches and their suitability, the presentation of the findings, the discussion and conclusions derived from the studies that will be included in the scoping review. Doing so is critical for the identification of any form of prejudice in the report of articles that will be included in the review.

#### Expected outcome

Because of the identified need to concentrate effort on the rehabilitation of individuals with schizophrenia, the investigators are interested in knowing the scientific work that has been performed in terms of the psychosocial rehabilitation of people living with schizophrenia in Sub-Sahara Africa. The scoping review can reveal the gap in rehabilitation in Sub-Saharan Africa and provide information on available strategies. This scoping review will form part of the research questions of the main study that the investigators plan to conduct in the most populous country in Sub-Sahara Africa, Nigeria.

## Abbreviations

PSR: Psychosocial rehabilitation
PCC: Population concept context
MMAT: Mixed Method Appraisal Tool
PRISMA: Preferred Reporting Items for Systematic Reviews and Meta-Analyses
